# Carfilzomib: A Promising Proteasome Inhibitor for the Treatment of Relapsed and Refractory Multiple Myeloma

**DOI:** 10.3389/fonc.2021.740796

**Published:** 2021-11-10

**Authors:** Shansa Pranami E. Jayaweera, Sacheela Prasadi Wanigasinghe Kanakanamge, Dharshika Rajalingam, Gayathri N. Silva

**Affiliations:** Department of Chemistry, Faculty of Science, University of Colombo, Colombo, Sri Lanka

**Keywords:** multiple myeloma, proteasome inhibitors, carfilzomib, anti-tumor effect, combination therapy

## Abstract

The proteasome is crucial for the degradation of intracellular proteins and plays an important role in mediating a number of cell survival and progression events by controlling the levels of key regulatory proteins such as cyclins and caspases in both normal and tumor cells. However, compared to normal cells, cancer cells are more dependent on the ubiquitin proteasome pathway (UPP) due to the accumulation of proteins in response to uncontrolled gene transcription, allowing proteasome to become a potent therapeutic target for human cancers such as multiple myeloma (MM). Up to date, three proteasome inhibitors namely bortezomib (2003), carfilzomib (2012) and ixazomib (2015) have been approved by the US Food and Drug Administration (FDA) for the treatment of patients with relapsed and/or refractory MM. This review mainly focuses on the biochemical properties, mechanism of action, toxicity profile and pivotal clinical trials related to carfilzomib, a second-generation proteasome inhibitor that binds irreversibly with proteasome to overcome the major toxicities and resistance associated with bortezomib.

## 1 Introduction

Multiple myeloma (MM) is a plasma cell neoplasm characterized by skeletal or bone damage due to the infiltration of bone marrow by the malignant plasma cells and the presence of abnormal monoclonal proteins (also known as “M proteins”) in serum and/or urine (1, 2). In the United States, MM accounts for approximately 1.8% of all the reported cancer cases (3, 4). Treatment of MM has advanced rapidly over the last two decades with the discovery of proteasome inhibitors and immunomodulatory agents (IMiD) as single agents and in combination therapy, drastically improving survival outcomes. Currently, several classes of chemical agents are available as therapeutic options for both newly diagnosed and relapsed and/or refractory MM (R/RMM) including proteasome inhibitors, IMiDs, histone deacetylase inhibitors, monoclonal antibodies, alkylators and steroids (2, 4).

Proteasome is a protease complex that mediates a number of cellular mechanisms through the maintenance of optimal levels of intracellular proteins required for cell cycle progression, cell apoptosis, and normal cellular processes *via* ubiquitin-dependent or ubiquitin-independent degradation of proteins (5–7). Inhibition of proteasomes results in the induction of cell cycle arrest and apoptosis *via* modulation of several pathways including stabilization of p53, activation of C-Jun NH2-terminal kinase (JNK), and deactivation of nuclear factor kappa-B (NFκB) leading to activation of both intrinsic and extrinsic caspase cascades. Besides, inhibition of proteasome can result in the accumulation of unfolded proteins in endoplasmic reticulum (ER), subsequently activating the Unfolded Protein Response (UPR) pathway leading to apoptosis (8).

Bortezomib, the first FDA approved proteasome inhibitor in the treatment of R/RMM, has shown a significant improvement in clinical results. Although bortezomib is a potent antineoplastic agent that targets the proteasome, its significant toxicities and resistance to some cancer cells has restricted its usage. As a result, a second-generation proteasome inhibitor, carfilzomib was developed with improved efficacy and safety profiles. In contrast to bortezomib which forms a reversible complex with the proteasome, carfilzomib irreversibly binds with the proteasome and inhibits its chymotrypsin-like activity. Carfilzomib also demonstrates an improved safety profile due to its specificity towards the proteasome’s chymotrypsin-like activity and rapid extrahepatic clearance of the free drug (9). In addition, the ability to penetrate almost all tissue types makes it a universal proteasome inhibitor that is effective in all tissues except those in the brain (Carfilzomib does not readily cross the blood-brain barrier) (10). Carfilzomib which is chemically an epoxyketone, was first approved by the FDA in 2012 as a single agent for the treatment of MM in patients subjected to at least two prior therapies including bortezomib and an IMiD, and demonstrated disease progression on or within 60 days after the last therapy (11). Later, carfilzomib was approved by the FDA in combination with dexamethasone or with lenalidomide and dexamethasone for the treatment of R/RMM. In August 2020, the FDA approval was obtained for the use of carfilzomib in combination with daratumumab, a human immunoglobulin Gκ (IgGκ) monoclonal antibody and dexamethasone in patients with relapsed or refractory MM who have received 1-3 prior lines of therapy (12).

This review provides a detailed report on the second-generation proteasome inhibitor carfilzomib including its mechanism of action and the pivotal clinical trials that have led to being granted FDA approval for use in monotherapy or combination therapy against R/RMM. Ixazomib, the latest FDA-approved proteasome inhibitor after carfilzomib, is also discussed briefly.

## 2 Progression of Relapsed and/or Refractory Multiple Myeloma (R/RMM)

Malignant plasma cells and their production of monoclonal proteins and cytokines are the primary causes of the clinical manifestations associated with MM, including end-organ damages such as hypercalcemia, renal insufficiency, anemia, and/or bone disease with lytic lesions, collectively known as CRAB features (1, 13). Recurring infections is another complication associated with MM due to their substantial effect on normal immune functions. Monoclonal gammopathy of undetermined significance, a premalignant plasma cell disorder precedes the progression of MM in almost all patients with or without the intermediate stage of an asymptomatic plasma cell proliferative disorder referred to as Smoldering multiple myeloma (14–16). In most patients with MM, relapse is inevitable partly due to the change in tumor biology and as each relapse typically occurs more aggressively leading to a treatment-refractory disease (17, 18). According to Dimopoulous et al. (17), three main patient groups were identified in R/RMM, namely, relapsed but not refractory, primary refractory, and relapsed and refractory. The relapsed but not refractory patient population is defined as “patients with active disease who have received one or more prior therapies and whose disease is not refractory to the most recent treatment” (17). The relapsed and refractory (RR) group is defined as “patients with disease relapse who have achieved minimal response (a reduction in serum or urinary M-protein >25%) or with the progressive disease while on salvage therapy or disease progression within 60 days of last therapy”. “Patients with primary refractory progressive disease and patients who do not achieve minimal response or better, including non-responding but non progressing patients who have no significant change in M-protein levels and no evidence of clinical progression” are included in the primary refractory group (17). Initially, the term “treatment-refractory” was more generic but with the advancement of treatment options for MM, it has become more specific in the context of agents used in therapy such as bortezomib and lenalidomide (19).

## 3 Proteasome Dependent Protein Degradation

Proteolysis is an essential process in cellular protein homeostasis where proteasomal degradation of intracellular proteins occurs predominantly through the ubiquitin-dependent pathway while some degradation goes through a ubiquitin independent pathway. These pathways are not mutually exclusive; the same protein can be degraded through either pathway depending on the cellular stress and the structure of the proteasome.

Ubiquitin-dependent proteasomal degradation is the major route of degradation for more than 80% of the intracellular proteins including proteins involved in apoptosis, cell survival, cell-cycle progression, DNA repair, and antigen presentation. This process consists of 3 steps; polyubiquitination, deubiquitination and proteasomal degradation of the target protein (7). Proteins targeted by Ubiquitin-Proteasome Pathway (UPP) are initially tagged by polyubiquitin molecules through a series of enzymes. Tagged proteins once recognized by the proteasome undergo deubiquitination by the regulatory subunits of proteasome followed by proteasomal degradation. Ubiquitin-dependent degradation is an energy-dependent process where ATP hydrolysis is needed for the activation of E1 enzyme (20, 21). Deregulation of the ubiquitin-dependent pathway impairs protein homeostasis and has been implicated in oncopathogenesis (22). For instance, the proteasome is a rational target in MM as malignant plasma cells that secret a large-amount of immunoglobulins (IgG) are highly dependent on the ubiquitin-dependent pathway for their survival (23).

The constitutive proteasome or 26S proteasome consisting of the 20S core structure and the 19S or 11S regulatory structure is found in the nucleus and cytoplasm of all eukaryotic cells and plays a major role in deubiquitination and proteolysis. 20S is a barrel-like catalytic core unit made up of four stacked rings (2 exterior and 2 interior rings) with a central channel where proteolysis occurs. Each exterior ring is composed of seven non-identical α subunits (α1-7) providing structural support and each interior subunit is composed of seven non-identical β subunits (β1-7), mainly chymotrypsin-like (β5), caspase-like (β1), and trypsin-like (β2) catalytic subunits that have the proteolytic activity (24). The 19S regulatory unit is responsible for processing the polyubiquitin chains which ensures that only targeted proteins are degraded through the proteasome (7). Immunoproteasome is the alternative form of 26S proteasome where the 19S unit is replaced by the 11S regulatory unit and β subunits are replaced by β5i, β1i and β2i which are engaged in mediating the immune response *via* antigen processing to produce antigenic peptides for the major histocompatibility complex (MHC) class 1. The expression of immunoproteasome is tissue-specific, and can be predominantly found in lymphoid tissues and hematopoietic cells (25).

## 4 Proteasome as a Potential Therapeutic Target for Cancers

Proteasomes play a vital role in all processes of the cell cycle including DNA replication, DNA repair, mitosis, apoptosis and maintaining signalling cascades *via* choreographed degradation of cyclin dependent kinases (CDKs). Additionally, proteasomes show significant involvement in normal cellular functions including signal transduction, stress response as well as in the degradation of misfolded and mutated proteins (26). Hence, proteasome has been identified as a highly attractive therapeutic target for cancers as diverse pathways are affected by proteasome inhibition that can contribute to anti-tumor effects.

**Figure 1:**
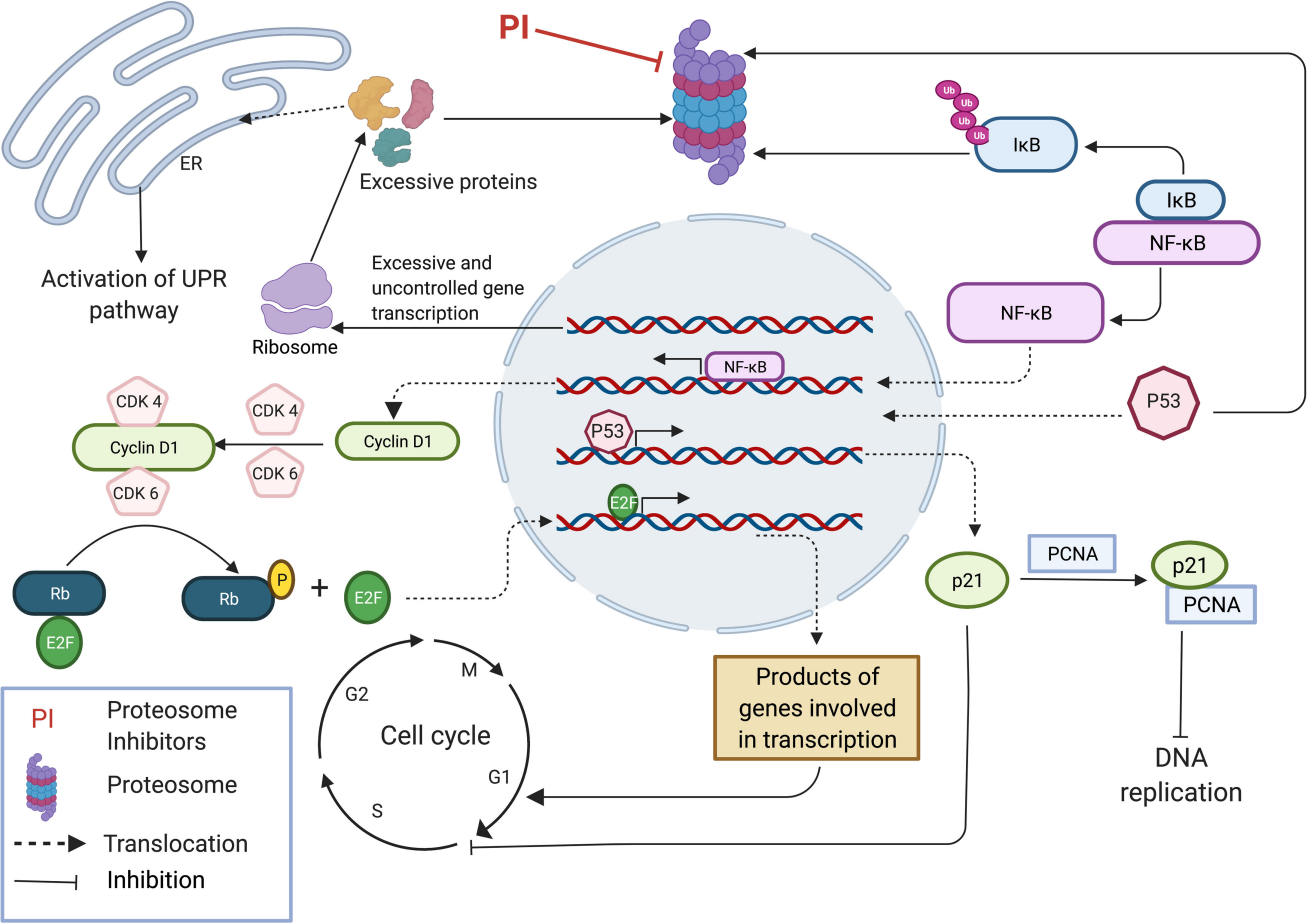
Proteasome inhibition modulates multiple regulatory pathways to induce anti-tumor effect. Accumulation of unfolded and mutated proteins in cancer cells due to uncontrolled gene transcription results in ER stress leading to the activation of UPR pathway. Inhibition of NFkB pathway by inhibiting the degradation of IkB inhibitor results in inhibition of many pro-survival pathways that induce cell cycle progression. Stabilization of p53 results in expression of p21 subsequently inhibiting DNA replication by binding with PCNA and inhibits cell cycle progression by interacting with CDKs. This image was created with BioRender (https://biorender.com/).

Excessive protein synthesis in tumor cells give rise to a plethora of abnormal and misfolded proteins as a consequence of genome mutations such as large duplications, deletions, translocations, inversions, altered number of chromosomes and unregulated transcription and translation (27, 28). The ER which plays a major role in folding and assembling of translated proteins has its own quality control mechanism by which it monitors misfolded or malformed proteins and targets them for degradation by UPP. Thus, in cancer cells, proteasome inhibition can lead to a build-up of a plethora of polyubiquitinated, misfolded cellular proteins causing ER stress, leading to the activation of the UPR pathway (**Figure 1**). UPR activates intracellular signal transduction, thereby maintaining homeostasis of ER by reducing protein synthesis (26, 29). Additionally, depending on the severity of ER stress, UPR arrests the cell cycle by upregulation of pro-apoptotic programs and simultaneously suppressing antiapoptotic enzymatic cascades resulting in ER stress-induced apoptosis (26, 30–35). Unlike normal cells, tumor cells are heavily dependent on proteasomes, the main component of the final step of protein degradation by UPP to remove unusual overproduction of malformed proteins and to prevent cell death (36, 37). MM is delineated as overproduction of IgG in plasma cells, which shows an elevated level of proteasome activity making them more vulnerable to proteasome inhibition compared to normal cells (24, 26, 38, 39).

The NFκB pathway, a pro-survival pathway is also affected by proteasome inhibition. NFκB is a transcription factor which induces the expression of a wide range of genes involved in cell proliferation, apoptosis and angiogenesis, and is generally considered a tumor promoting factor (27, 40, 41). NFκB normally exists in an inactive state in the cytoplasm because of IκB (inhibitor of NF-κB) which is an endogenous inhibitor of NFκB. In response to different stimuli, IκB kinase gets activated and phosphorylates IκB leading to its ubiquitination and degradation by proteasome (27, 42). Degradation of IκB allows the activation of heterodimer P50/P65 NFκB transcription factors, allowing them to translocate into the nucleus. IκB accumulates due to proteasome inhibition, resulting in an inactive NFκB complex which is unable to translocate into the cell nucleus to stimulate the survival and progression of MM cells (26, 43) (**Figure 1**). Mammals express five different types of NFκB proteins namely RelA (p65), RelB, C-Rel, p50 and p52. Proteasomes are involved in the maturation process of p50 and p52 NFκB proteins which are initially synthesized as large precursors. Hence, proteasome inhibition also blocks NFκB pathway by affecting the maturation process of the two main NFκB proteins (27, 44). Expression of Cyclin D1 is regulated under the NFκB pathway and plays a major role in cancer progression by acting as the key regulator of the late G1 phase of the cell cycle. Cyclin D1 complexes with CDK4/6 and generates a phosphorylated form of Rb (Retinoblastoma) protein allowing the release of E2F transcription factor inducing its activation. Released E2F induces the expression of Cyclin E which then interacts with CDK2 resulting in hyper regulation of Rb, expression of Cyclin A and genes involved in DNA synthesis, leading to the progression of the cell cycle to S phase (27, 45–48) (**Figure 1**). At first, it was thought that the anti-tumor effect by proteasome inhibition resulted from the inhibition of NFκB pathway, as this pathway is involved in several most important processes of tumor growth including cell proliferation, metastasis and angiogenesis. However, cellular toxicity profile of proteasome inhibition was not reproduced on employing a potent IκB kinase inhibitor which acts in a similar manner to the proteasome inhibitors, i.e by blocking the NFκB activation. This experiment demonstrates that the inhibition of other pathways is equally important to generate an effective anti-tumor effect (26).

Proteasome inhibition also mediates other pro-apoptotic effects in cells such as stabilization of Bim (bcl-2-interacting mediator of cell death), Bid (BH3-interacting domain death agonist), Bik (Bcl-2-interacting killer) etc. subsequently activating pro-apoptotic effector compounds including Bax (BCL2 Associated X, Apoptosis Regulator) and Bak (Bcl-2 homologous antagonist/killer) (30, 49–51). Generally, the cellular concentrations of pro-apoptotic family proteins Bim, Bid and Bik are regulated by UPP. Inhibition of proteasome leads to accumulation of these proteins in the cell, resulting in caspase activation and apoptosis (26, 52, 53). Furthermore, proteasome inhibition induces the expression, phosphorylation and accumulation of p53 (26, 30, 54), along with stabilization of p21 and p27 (30, 55) all of which are cyclin dependent kinase inhibitors, leading to cell cycle arrest and inhibition of cell proliferation. Primarily pro-apoptotic features of p53 play a prominent role in tumor suppression, regulating DNA repair, apoptosis and senescence. Stabilization of p27 by proteasome inhibition is important as it suppresses the activity of CDK2/Cyclin E and CDK2/Cyclin A complexes mediating progression to the G1 phase and suppressing transition to the S phase (27, 56). P21 binds with the CDK2/Cyclin E complex inhibiting the onset of the S phase in the cell cycle and also binds with CDK1/Cyclin B complex resulting in cell cycle arrest in the G2 phase. Furthermore, p21 inhibits DNA replication by binding with proliferating cell nuclear antigen (PCNA). Under normal conditions, p21 levels are largely controlled by p53 (**Figure 1**). Thus, stabilization of p53 by proteasome inhibition results in high levels of p21 leading to negative regulation of the cell cycle (27, 57–60).

Additionally, agents that inhibit proteasomes are known to activate JNK triggering upregulation of Fas (30, 61) leading to programmed apoptotic cell death by activation of caspase 8 and 3 (26, 55). Noxa is a pro-apoptotic member of the Bcl-2 family that interacts with p53 in response to stimuli such as hypoxia, cytokine signaling or mitogenesis leading to apoptosis. Under normal conditions, Noxa is rapidly degraded by proteasome and accumulation of Noxa due to proteasome inhibition results in activation of caspase 9 (26, 49, 62). Proteasome inhibition also induces the production of reactive oxygen species, causing mitochondrial injury, resulting in release of pro-apoptotic compounds such as cytochrome C (30, 63). Furthermore, inhibition of proteasome leads to reduced levels of IGF-1 and IGF-1R, suppressing the activation of NFκB and other antiapoptotic proteins including Akt (Protein kinase B), FADD-like IL-1β-converting enzyme inhibitory protein, survivin resulting in cell cycle arrest and apoptosis (34, 64–66). Proteasome inhibitors also downregulate cell adhesion molecules and secretion of cytokines (27).

As mentioned, proteasome inhibition suppresses cancer progression and growth by interfering with different pathways such as activation of UPR pathway, downregulation of NFκB pathway and stabilization of p53 signaling (27). Proteasome inhibition is also associated with several pro-survival effects. However, when considering the overall downstream effects of proteasome inhibition and dependence of cancer cells on the ubiquitin protease system and its sensitivity to ER stress, it can be suggested that proteasome inhibition has immense potential as a therapeutic target for cancers (30, 63).

Bortezomib, the first FDA approved (2003) in-class proteasome inhibitor, a dipeptide boranate which can slowly bind to the catalytic site of the 26S proteasome (67), has shown positive clinical responses and outcomes.

In MM patients, bortezomib has prolonged progression- free survival significantly compared to previous conventional chemotherapeutic agents such as alkylating agents or vincristine, doxorubicin and dexamethasone (VAD) (68).

## 5 Carfilzomib: A Promising Antineoplastic Drug Against R/RMM

Even though bortezomib is a potent inhibitor of the proteasome and has prominent antitumor activity, its significant toxicities and resistance have restricted its usage. As a result, research efforts have been made to develop second-generation proteasome inhibitors with more efficacy, improved safety profile and more convenient administration methods to broaden the range of anti-tumor therapy options to overcome resistance by cancer cells. Carfilzomib (formerly PR-171) is a second-generation proteasome inhibitor which is chemically, a tetrapeptide epoxyketone analogue derived from epoxomicin, a natural product isolated from actinomyces (8, 10). Carfilzomib (Kyprolis^®^, developed by Proteolix/Onyx Pharmaceuticals and available through Amgen) (11, 69) was approved by the FDA in July, 2012 to be used as a single agent for the treatment of MM in patients with refractory disease, specifically for patients who had received at least two prior lines of therapy and have shown disease progression on or within 60 days of completion of the last therapy (11, 24, 26, 70). The chemical name of carfilzomib is (2S)-N-((S)-1-((S)-4-methyl-1-((R)-2-methyloxiran-2-yl)-1-oxopentan-2-ylcarbamoyl)-2 phenylethyl)-2-((S)-2-(2-morpholinoacetamido)-4 phenylbutanamido)-4-methylpentanamide. The molecular formula is C_40_H_57_N_5_O_7_, and the molecular mass is 719.91 (11).

FDA approved Carfilzomib is used as a formulation (Kyprolis) which includes other chemical substances such as Sulfobutylether beta-cyclodextrin, Citric acid and Sodium Hydroxide for pH adjustments additionally to the active pharmaceutical ingredient Carfilzomib. Cyclodextrin in Kyprolis is important for improving aqueous solubility of Carfilzomib by forming inclusion complexes (71). However, recent studies have shown improved injection formulas for Carfilzomib with organic acids and nanoparticles to reduce the pharmaceutical limitations of Kyropolis, such as low solubility, poor stability and short release of half-life which can enhance the use of carfilzomib for the treatment of multiple myeloma as well as solid tumors (72, 73).

Similar to bortezomib, carfilzomib inhibits chymotrypsin-like activity of the proteasome by binding to the hydroxyl and amino groups of the N-terminal threonine of the β5 subunit in 20S proteasome. However unlike bortezomib which is also a potent inhibitor of caspase-like activity, carfilzomib, at therapeutic concentrations, does not effectively inhibit trypsin or caspase-like activity but, reduces chymotrypsin-like activity by more than 80% by inhibiting the β5 subunit of the constitutive proteasome (c20S) and LMP7 (β5i) subunit of immunoproteasome (i20S) (30, 74). Additionally, carfilzomib is a highly selective proteasome inhibitor with minimum effect to other non-proteasome substrates such as serine proteases, cathepsin G, cathepsin A, rennin, dipeptidyl peptidase II, and mitochondrial serine protease HtrA2/Omi which are affected by bortezomib. Peripheral neuropathy, the major side effect of Bortezomib may be due to the inhibition of HtrA2/Omi; a compound known to be involved in neuronal survival (75).

**Figure 2:**
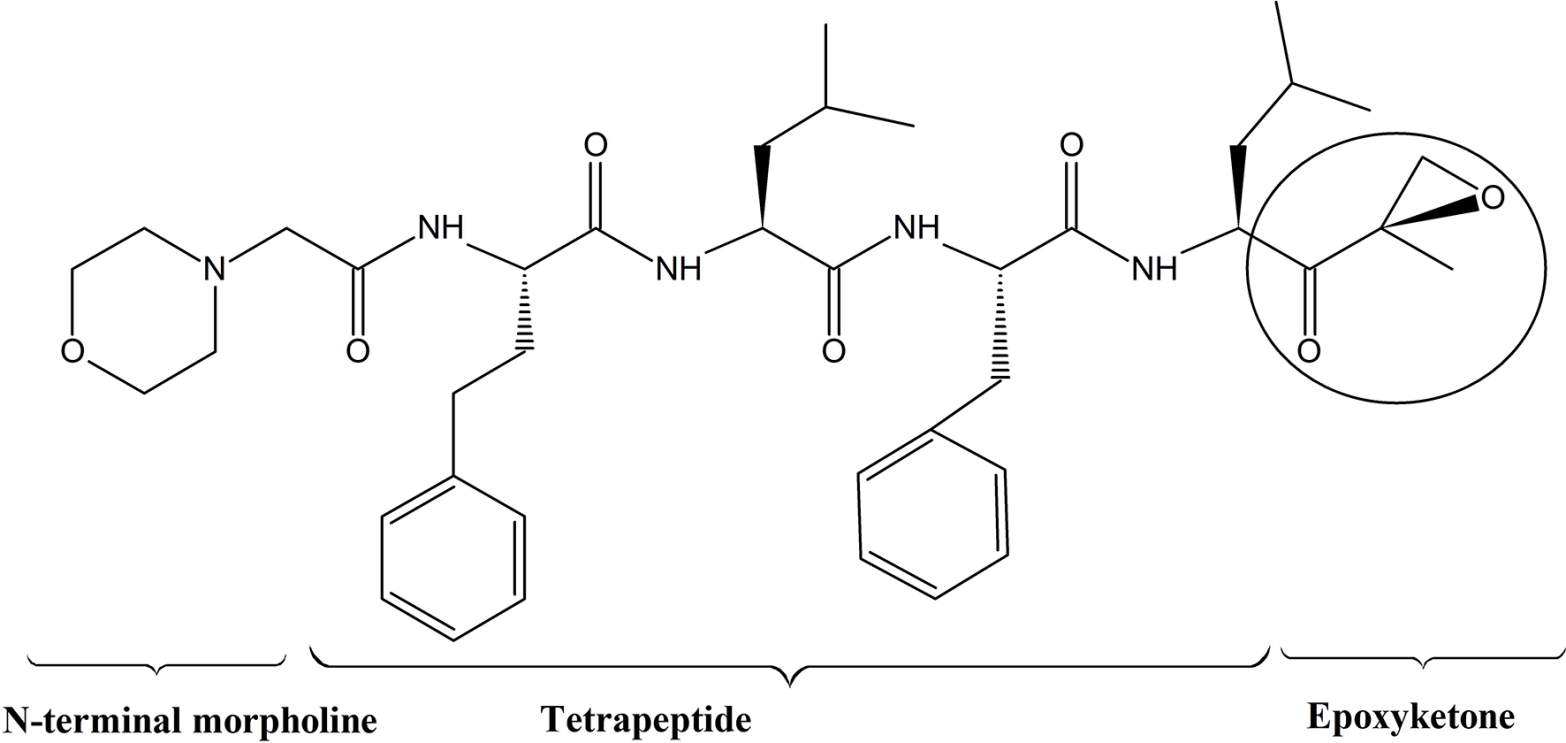
Structure of carfilzomib; Carfilzomib contains a tetrapeptide portion and an epoxyketone pharmacophore (11).

**Figure 3:**
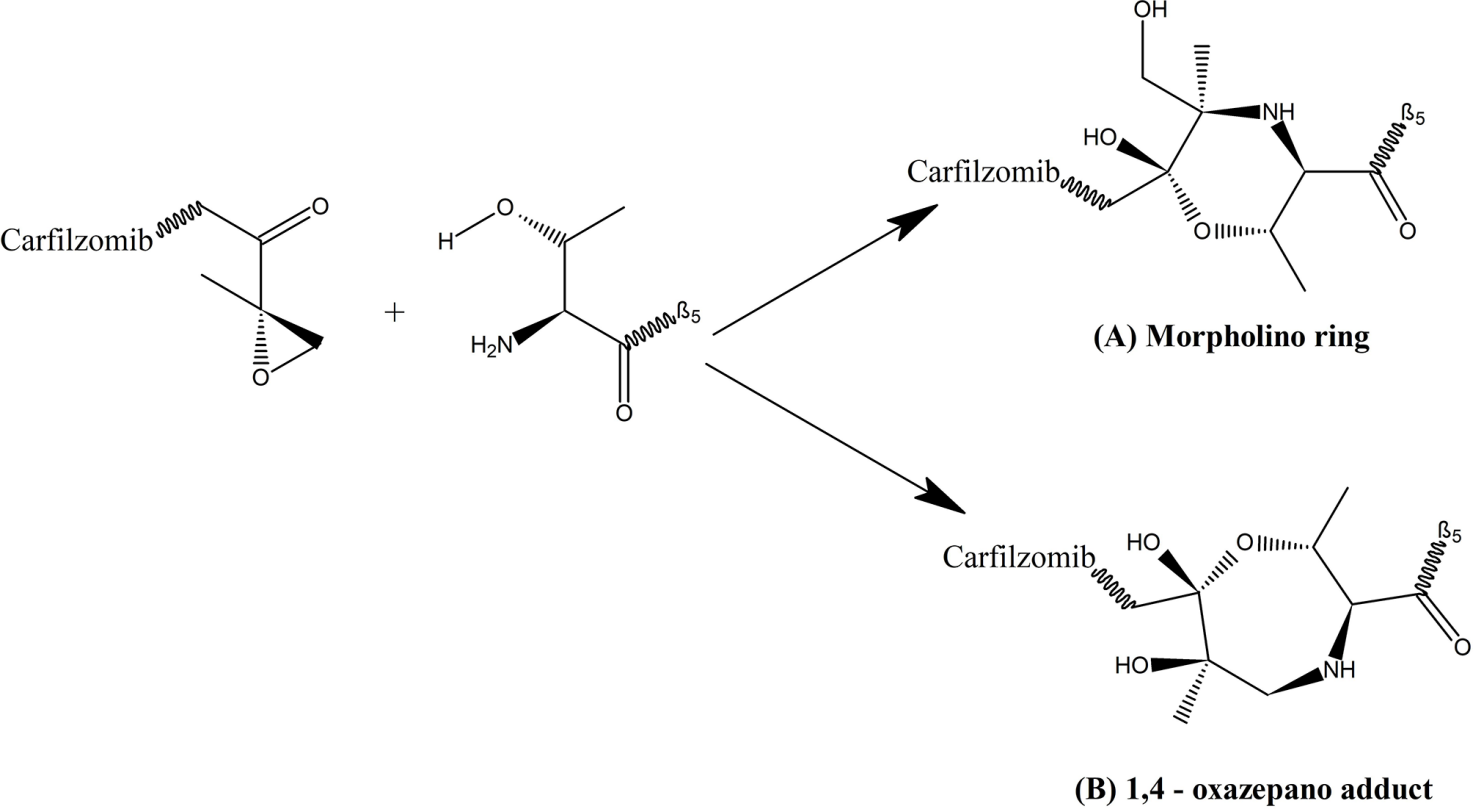
Complex formed between epoxyketone pharmacophore of carfilzomib and the catalytic threonine of β5 subunit of proteasome (79); **(A)** Morpholino ring which is a six membered ring formed by bonding of Thr1Oγ nucleophile of β5 subunit with α carbon of the epoxyketone pharmacophore, proposed by Groll et al. (77); **(B)** 1, 4- oxazepano adduct which is a seven membered ring formed by bonding of Thr1Oγ nucleophile of β5 subunit with β carbon of the epoxyketone pharmacophore proposed by Schrader et al. (78).

Epoxymicine and its analogues including carfilzomib contain two key elements; a peptide portion and an epoxyketone pharmacophore. The peptide portion selectively and tightly binds with substrate binding pockets of the proteasome, and the epoxyketone pharmacophore irreversibly inhibits the activity of the β5 subunit of proteasome by stereospecifically interacting with the catalytic threonine residue. This reaction is unique to proteasomes, as they utilize the sidechain hydroxyl group of an NH_2_ terminal threonine of the β5 subunit as the catalytic nucleophile. Furthermore, the N-terminal morpholine in carfilzomib increases its aqueous solubility to make it a potent drug for the treatment of MM (76) (**Figure 2**). According Groll et al. *(* 77 *)*, formation of dual covalent bonds between the epoxyketone pharmacophore and proteasome results in a six-membered morpholino ring. In contrast, a recent study carried out by Schrader et al. (78), demonstrated that the epoxyketone pharmacophore forms a seven membered 1, 4- oxazepano-ring adduct with the β5 subunit of the proteasome *via* dual covalent bonds; one between the C-terminal ketone moiety of carfilzomib and the catalytic Thr1Oγ nucleophile of the β5 subunit and a second covalent bond between carfilzomib’s epoxide β carbon and the adjacent Thr1N-terminal amino group of the β5 subunit. Basically, in contrast to the oxazepano-ring adduct, in the morpholino ring the Thr1Oγ nucleophile of the β5 subunit forms a bond with the α carbon of the epoxyketone pharmacophore instead of the β carbon (**Figure 3**). Formation of dual covalent bonds results in a unique mechanism requiring close juxtaposition of side-chain hydroxyl and amino groups of the catalytic N terminal threonine of β5 subunit (26, 27, 30, 69, 78, 80). This extraordinary inhibiting mechanism of carfilzomib makes it comparatively less toxic and specific towards proteasomes than bortezomib and other proteasome inhibitors. When considering the proteasome-inhibitor complexes, although both bortezomib and carfilzomib form covalent adducts with their substrates, their hydrolytic stability varies. The 1, 4- oxazepano adduct formed between carfilzomib and the proteasome is an irreversible complex. In contrast, bortezomib forms a slowly reversible tetrahedral intermediate with the proteasome. Although carfilzomib irreversibly inhibits proteasomes, the rate of proteasome recovery is not significantly different with both drugs. The high rate of proteasome recovery can be due to increased mRNA transcription and *de novo* proteasome synthesis (9).

In normal cells, selective inhibition of the chymotrypsin-like activity of the 20S proteasome yields a minor effect on total protein degradation (81). Unlike normal cells, hematologically derived tumor cells express both types of proteasomes; constitutive proteasome and immunoproteasome. Selective and simultaneous inhibition of both β5 and β5i (LMP7) subunits of the proteasome and immunoproteasome by carfilzomib, induces an impressive anti-tumor response in MM compared to single subunit inhibition. Surprisingly, carfilzomib causes the least cytotoxic effects to normal cells. The specificity of carfilzomib towards β5 and β5i (LMP7) subunits of the proteasome and immunoproteasomes respectively, is a critical feature of its low toxicity as the inhibition of all subunits of the proteasomes can lead to detrimental effects in normal cells.

Selective inhibition of both β5 and β5i subunits results in accumulation of proteins in tumor cells leading to the initiation of UPR *via* induction of ER stress response. Inhibition of the chymotrypsin-like activity of the proteasome also induces p53-mediated apoptosis by stabilization of Noxa (74). Furthermore, the mechanism of cell death by carfilzomib involves the activation of common effector caspase-3 *via* both intrinsic and extrinsic pathways. Additionally, inhibition of proteasome activity leads to the activation of JNK which will eventually cause C-Jun phosphorylation and cleavage of polyADP ribose (30). Compared to bortezomib, carfilzomib increases the levels of caspase-3, caspase-8 and caspase-9 by 1.5, 1.8 and 2.0 folds respectively (8, 34). Intravenous administration of carfilzomib is effective in a vast variety of body tissues due to its potential penetration ability throughout the body displaying a universal proteasomal inhibition. However, carfilzomib, is unable to effectively cross the blood-brain barrier making it less potent for the brain (8, 10). The liver is also relatively insensitive to carfilzomib despite of its ability to penetrate the liver. The possible reason for this is the competition between carfilzomib metabolizing enzymes and proteasomal active sites (30, 82).

Carfilzomib is well-tolerated in patients and has a half-life of ~20 minutes. It readily metabolizes into toxicologically insignificant metabolites (displays a high plasma clearance of 195-319 ml/(min. kg) in rats) which is primarily mediated by extrahepatic metabolism (30, 82). Unlike most drugs, carfilzomib is not metabolized in the liver, reducing widespread toxic effects (75, 82). Carfilzomib is rapidly metabolized into inactive peptides and/or diol compounds in the plasma, primarily *via* peptidase cleavage and epoxide hydrolysis (11). Furthermore, in contrast to bortezomib, carfilzomib generates less hepatic cytochrome P450 – dependent oxidative metabolites (30, 80, 83, 84). Metabolites of carfilzomib are partially excreted *via* the biliary and renal systems. However, a portion of the protein backbone of the drug is thought to be degraded and utilized by the host’s anabolic pathways because of radioactive H-carfilzomib has been detected in the body long after a single dose (>44% - 168 hours post dose) although the majority is excreted within 4 hours of dosing (9, 30).

However, rapid clearance from blood does not reduce the potency of carfilzomib because of its irreversible binding to the 20S proteasome. Hence, even a brief exposure is sufficient to provide prolonged inhibition of proteasomes, even after the free drug has been metabolized and cleared (30). Additionally, the short half-life is crucial for minimizing potential drug related side-effects caused by exposure and binding to non-specific targets (82). Although, the exact mechanism of action is unknown, carfilzomib overcomes the proteasome inhibitor resistance in several bortezomib-resistant MM cell lines (8, 11). This could be due to the irreversible binding of carfilzomib to the chymotrypsin-like catalytic site delaying the recovery of proteasome activity (30). Despite these therapeutic advances, carfilzomib resistance was observed in some patients probably due to the overexpression and mutations of proteasome catalytic subunits. Overexpression of efflux pump P-glycoprotein (P-gp) can also result in low levels of intracellular carfilzomib as carfilzomib is identified as a substrate for P-gp (27, 85–87).

## 6 Clinical Evaluations of Carfilzomib in R/RMM

Carfilzomib has shown increased efficacy compared with bortezomib in both preclinical and clinical settings (8, 88). For instance, MM cells were more sensitive to carfilzomib compared to bortezomib and accordingly, carfilzomib was more potent in inducing apoptosis compared to bortezomib. Additionally, carfilzomib was active against MM cells resistant to bortezomib (8). Carfilzomib in combination with dexamethasone, has shown significantly longer progression free survival and overall survival than bortezomib in combination with dexamethasone in phase III ENDEAVOR trial and interim analysis. Such that, these preclinical and clinical data established carfilzomib to be a more potent inhibitor of MM and set the stage to follow up clinical trials testing the efficacy of several carfilzomib treatment regimens. Key clinical trials involving carfilzomib as a single agent and in combination with other agents for R/RMM are discussed in this review with regards to efficacy and toxicity. Some of the pivotal clinical trials utilizing Carfilzomib regimens against R/RMM are shown in **Table 1**.

**Table 1:** Details of the pivotal clinical trials utilizing carfilzomib regimens against R/RMM.

Trial	Phase	Number of Patients (n)	Carfilzomib Dose	ORR	Median DOR (Months)	Median PFS (Months)	Median OS (Months)
PX-171-003 (89)	II	266	20 mg/m^2^(first cycle) 27 mg/m^2^ (cycle 2 and beyond)	23.7%	7.8	3.7	15.6
PX-171-004 (90)	II	Cohort 1 (n=59)	Cohort 1	42.4% *vs*. 52.2%	13.1 *vs*. not reached	8.2 *vs*. not reached	not reported
			20 mg/m2(for all cycles)				
Cohort 1 *vs*. cohort 2		Cohort 2 (n=70)	Cohort 2				
			20 mg/m2(first cycle)				
			27 mg/m2 (cycle 2 and beyond)				
FOCUS (91) (Carfilzomib group *vs*. control group)	III	Carfizomib group (n=157)	Carfilzomib group	19.1% *vs*. 11.4%	7.2 *vs*. 9.5	3.7 *vs*. 3.3	10.2 *vs*. 10.0
			20 mg/m^2^(first cycle; days 1&2)				
		Control group (n= 158)	27 mg/m^2^ (all subsequent dosing days and cycles)				
ASPIRE (92, 93)	III	792	KRd group	87.1% *vs*. 66.7%	28.6 *vs*. 21.2	26.3 *vs*. 17.6	48.3 *vs*. 40.4
(KRd *vs*. Rd)		KRd (n=396)	20 mg/m^2^(first 2 doses)				
		Rd (n=396)	27 mg/m^2^ (dose 3 and beyond)				
ENDEAVOR (88, 94)	III	929	Kd group	77% *vs*. 63%	21.3 *vs*. 10.4	18.7 *vs*. 9.4	47.6 *vs*. 40.0
(Kd *vs*. Vd)		Kd (n=464)	20 mg/m^2^(first 2 doses)				
		Vd (n=465)	56 mg/m^2^ (dose 3 and beyond)				
A.R.R.O.W (95) (once weekly carfilzomib *vs*. twice weekly carfilzomib)	III	478	Once weekly group	62.9% *vs*. 40.8%	15.0 *vs*. 13.8	11.2 *vs*. 7.6	not reported
		once weekly (n=240)	20 mg/m^2^ (first cycle; day1)				
			70 mg/m^2^ (all subsequent dosing days and cycles)				
		twice weekly (n=238)	Twice weekly group				
			20 mg/m^2^ (first cycle; days 1 & 2)				
			27 mg/m^2^ (all subsequent dosing days and cycles)				
#NCT01998971 (96)	Ib	85	Once weekly	84%	not reported	not reached	not reached
			20 mg/m^2^ (first dose)				
			70 mg/m^2^ (all subsequent dosing days and cycles				
CANDOR (97) DKd *vs*. Kd	III	466	All the patients twice weekly	84% *vs*. 75%	not estimable *vs*. 16.6	not reached *vs*. 15.8	not reached
		DKd (n=312)	20 mg/m^2^ (first cycle; days 1 and 2)				
		Kd (n=154)	56 mg/m^2^ (all subsequent dosing days and cycles				
IKEMA (98) IKd *vs*.Kd	III	302	All the patients twice weekly	87% *vs*. 83%	not reported	not reached *vs*. 19.15	not reported
		IKd (n =179)	20 mg/m2 (first cycle; days 1 and 2)				
		Kd (n= 123)	56 mg/m2 (all subsequent dosing days and cycles				

KRd, carfilzomib +Rd; Rd, lenalidomide + dexamethasone; Kd, carfilzomib + dexamethasone; Vd, bortezomib + dexamethasone; DKd, Daratumumab + Kd; IKd, Isatuximab + Kd; ORR, Overall Response Rate; DOR, Duration of Response; PFS, Progression Free Survival; OS, Overall Survival.

### 6.1 Carfilzomib Monotherapy

Based on the promising results observed in early phase I clinical studies including PX-171-001 and PX-171-002 trials (99, 100), several phase II studies concerning carfilzomib were initiated in patients with MM. Most importantly, two parallel phase II trials evaluating the efficacy of single agent carfilzomib in patients with R/RMM, PX-171-003 and PX-171-004 trials were initiated based on the data from the above phase 1 trials (100). PX-171-003 was designed to investigate the activity of carfilzomib in patients with R/RMM, who had received 1-2 prior lines of therapies and had also been exposed to bortezomib and immunomodulatory therapy (89, 101).

PX-171-004 was a phase II, open label, multicenter study which was originally designed to evaluate the impact of carfilzomib treatment in relation to bortezomib therapy in less heavily pretreated (received 1-3 prior lines of therapy) patients with R/RMM. Later, the study was amended to evaluate carfilzomib activity separately in bortezomib-naive patients with R/RMM. A patient population of 129 bortezomib-naive patients with R/R MM was separated into two cohorts. Patients in cohort 1 (n=59) received carfilzomib at a dose of 20 mg/m^2^ for all treatment cycles, whereas patients in cohort 2 (n=70) received carfilzomib at a starting dose of 20 mg/m^2^ for cycle 1 and an escalated dose of 27 mg/m^2^ for all subsequent cycles. The primary end point of the study was an overall response rate (ORR) of 42.4% in cohort 1 and 52.2% in cohort 2. Median duration of response (DOR) and median progression free survival (PFS) in cohort 1 were 13.1 and 8.2 months respectively and neither of these two values were reached in cohort 2. The clinical benefit response which includes both ORR and minimal response was 59.3% in cohort 1 and 64.2% in cohort 2. The most common treatment related adverse events (AEs) included fatigue (45.0%), nausea (41.9%), anemia (31.0%), dyspnea (27.9%), thrombocytopenia (23.3%) and neutropenia (22.5%). More than one third of the patient population was able to continue treatment for more than 12 months without significant toxicity and none of the patients discontinued treatment due to peripheral neuropathy (90).

Siegel et al. (89), carried out a phase II study for single agent carfilzomib in patients with R/RMM where the primary endpoint was ORR. In this PX-171-003-A1 trial, carfilzomib was given intravenously over 2-10 minutes on days 1,2,8,9,15 and 16 of each 28 days cycle for up to 12 cycles. Carfilzomib was dosed at 20 mg/m^2^ during the first cycle and the dose was increased to 27 mg/m^2^ in the subsequent cycles. To prevent potential infusion reactions, 4 mg of dexamethasone was given orally or intravenously prior to each dose of carfilzomib. Patients who had received at least 2 or more prior regimens for MM were considered for the study. A total of 266 patients with a median of 5 prior lines of therapy including bortezomib and an IMiD agent (lenalidomide and thalidomide) participated. Among them, 95% were refractory to their last therapy while 80% were double refractory to bortezomib and lenalidomide. Among the 257 response evaluable patients for drug efficacy, the reported ORR was 23.7% with a 37.0% clinical benefit response. Median DOR was 7.8 months while the median overall survival (OS) was 15.6 months. Only 15% of the patients completed the 12 cycles and the remaining population discontinued the therapy primarily due to progressive disease (59%) or AEs (12%) (89). Clinically significant data from this study regarding highly pretreated MM patients led to accelerated approval from FDA for carfilzomib monotherapy in 2012 for the treatment of MM patients who had at least two prior therapies including bortezomib and an IMiD and demonstrated disease progression on or within 60 days after the last therapy (11).

A phase III study was required by the European Medicines Agency in order to approve carfilzomib as a single agent against relapsed and refractory MM (RRMM). Hence a randomized, phase III, open label, multicenter study (FOCUS) was initiated to assess carfilzomib monotherapy against low dose corticosteroids with optional cyclophosphamide in patients with RRMM. A total number of 315 patients with heavily pretreated RRMM participated in this PX-171-011 (91) where the primary end point was the OS. Patients were randomized into a carfilzomib group (n=157) and a control group (n=158) receiving a low dose of corticosteroids with optional cyclophosphamide. The carfilzomib group received a starting dose of 20 mg/m^2^ of carfilzomib, intravenously for 10 minutes on days 1 and 2 of cycle 1 which was increased to 27 mg/m^2^ thereafter. Patients in the control group received a low dose of corticosteroids containing 6 mg dexamethasone or 30 mg prednisone every other day or another equivalent corticosteroid dose. In addition to corticosteroids an optional dose of 50 mg cyclophosphamide (maximum 1400 mg for a 28 days cycle) was given to 95% of the patients in the control arm. Both groups had a median of 5 prior regimens. Median OS was 10.2 *vs*. 10 months between the carfilzomib and the control group (hazard ratio (HR) = 0.975; *P* = 0.4172). Since no significant improvement in median OS was observed in the carfilzomib group compared to the control, the primary endpoint of the study was not reached. Median PFS was 3.7 months in the carfilzomib group compared to 3.3 months in the control group (HR 1.091; *P* = 0.2479). Overall response rate was higher in the carfilzomib treatment group (19.1%) compared to control group (11.4%), which was significant (*P* = 0.0305). Similarly, the number of patients achieving a minimal response or better was higher in the carfilzomib treatment group (31.2% *vs*. 20.8%). The most commonly observed grade 3 or higher AEs were anemia (25.5 *vs*. 30.7%), thrombocytopenia (24.2 *vs*. 22.2%) and neutropenia (7.6 *vs*. 12.4%) in carfilzomib group *vs*. control group respectively. Cardiac failure of any grade was observed in 7 patients in the carfilzomib group whereas only 1 patient was reported from the control group (91).

### 6.2 Carfilzomib in Combination Therapy

In this section, the key clinical trials which led to the FDA approval of carfilzomib plus dexamethasone and/or lenalidomide and carfilzomib plus daratumumab and dexamethasone (DKd) treatment regimens for the treatment of R/RMM are discussed in detail.

#### 6.2.1 Carfilzomib and Dexamethasone (Kd)

Clinical data obtained from phase I study (102) and a phase II study (103) evaluating the combination of carfilzomib and dexamethasone in patients with advanced MM, indicated that carfilzomib in combination with dexamethasone could be a promising treatment option for patients with R/RMM. This lead to the ENDEAVOR trial (94), a randomized, open label, phase III, multicenter study involving patients with relapsed or refractory MM who had undergone 1-3 prior line of therapies. The main objective of the study was to evaluate the combination of carfilzomib and dexamethasone against the combination of bortezomib and dexamethasone as a treatment option for relapsed or refractory MM. A total number of 929 patients with relapsed or refractory MM were randomly assigned to two groups; the carfilzomib and dexamethasone group (n=464) and the bortezomib and dexamethasone group (n=465). The carfilzomib group received carfilzomib at a starting dose of 20 mg/m^2^ on days 1 and 2 of cycle 1, followed by a dose of 56 mg/m^2^ as a 30 minute intravenous infusion in the subsequent cycles with a dexamethasone dose of 20 mg as oral or intravenous infusion. The bortezomib group received a 1.3 mg/m^2^ of bortezomib as an intravenous bolus or a subcutaneous injection and 20 mg of dexamethasone (oral or intravenous infusion).

The primary end point of the study was the PFS. A significant advantage in terms of PFS was observed in carfilzomib treated patients compared to the bortezomib group (median PFS 18.7 months *vs*. 9.4 months, HR 0.53; *P* < 0.0001). The ORR was 77% in the carfilzomib group compared to 63% in the bortezomib group (*P* < 0.0001). The median DOR was 21.3 months and 10.4 months in the carfilzomib and bortezomib groups respectively (94). In 2017, an updated OS analysis for the ENDEAVOR study was published which showed a significant improvement of OS with carfilzomib and dexamethasone compared to bortezomib and dexamethasone (median OS 47.6 *vs*. 40.0 months, *P* = 0.010). The most common grade 3 or worse AEs included anemia (14% *vs*. 10%), hypertension (9% *vs*. 3%), thrombocytopenia (8% *vs*. 9%), and pneumonia (7% *vs*. 8%). Occurrence of grade 2 or higher peripheral neuropathy was significantly higher in the bortezomib-treated group compared to the carfilzomib-treated group. Clinically significant improvements were observed in PFS, objective response rate and OS for the carfilzomib and dexamethasone treated group compared to the bortezomib and dexamethasone treated group (88). A *post hoc* analysis for this trial indicated a median PFS of 18.7 *vs* 6.6 months (HR 0.50, 95% confidence interval (CI) 0.36 - 0.68) and a median OS of 33.6 *vs*. 21.8 months (HR 0.75; 95% CI 0.56 - 1.00 between carfilzomib treated group and bortezomib group in frail patients with relapsed and/or refractory multiple myeloma (104). Based on clinical data from the ENDEAVOR trial, FDA approval was given for carfilzomib to be used in combination with dexamethasone for patients with R/RMM who have received 1-3 prior lines of therapies (75).

Initially carfilzomib was approved by the FDA for a twice-weekly schedule at a dose of 27 mg/m^2^ combined with lenalidomide and dexamethasone (KRd) or 56 mg/m^2^ plus dexamethasone (Kd) for patients with R/RMM. Since many patients may find it inconvenient to undergo the twice a week dosing schedule, improved dosing strategies were investigated. The once weekly carfilzomib dosing was studied in the preliminary phase 1/2 CHAMPION trial (105) which established the maximum tolerated dose at 70 mg/m^2^ in combination with dexamethasone. Findings from the CHAMPION trial led to the initiation of the randomized open label phase III A.R.R.O.W trial (95) comparing the PFS of once-weekly carfilzomib administration against a twice-weekly administration in patients with RRMM. A total of 478 patients were randomly assigned to receive once-weekly carfilzomib (30 min intravenous infusion on days 1, 8 and 15 of all cycles; 20 mg/m^2^ on day 1 of cycle 1 and 70 mg/m^2^ thereafter) or twice-weekly carfilzomib (10 min intravenous infusions on days 1, 2, 8,9,15 and 16; 20 mg/m^2^ on days 1 and 2 of cycle 1 and 27 mg/m^2^ thereafter). All the patients received 40 mg of dexamethasone on days 1,8,15 (all cycles) and 22 (cycles 1-9 only). The once-weekly group reported a higher median PFS compared to the twice-weekly group (11.2 months *vs*. 7.6 months, HR 0.69, *P* = 0.0029) (95). Data from the *post hoc* analysis conducted by Facon et al. (104), reported a median PFS of 10.3 *vs*. 6.6 months (HR 0.76, 95% CI 0.49 - 1.16) between once weekly group and twice weekly group in frail patients (104). Once-weekly carfilzomib schedule in combination with dexamethasone was approved for RRMM therapy by the US FDA in 2018 (106).

#### 6.2.2 Carfilzomib, Lenalidomide and Dexamethasone (KRd)

Promising results observed in the phase I and II studies (107, 108), PX-171-006 on carfilzomib with dexamethasone and lenalidomide against relapsed MM led to the phase III ASPIRE trial. In this randomized, multicenter study the combination of carfilzomib with dexamethasone and lenalidomide (carfilzomib group) and dexamethasone plus lenalidomide (control) were evaluated against R/RMM in patients who received 1 - 3 prior lines of treatments. A total number of 792 patients with a median number of 2 prior treatments were randomly assigned to either the carfilzomib group or the control group. Patients in the carfilzomib group received a starting dose of carfilzomib at 20 mg/m^2^ on days 1 and 2 of cycle 1; increased to 27 mg/m^2^ thereafter (on days 1, 2, 8, 9, 15, and 16 in a 28 days cycle) along with 25 mg of lenalidomide on days 1 through 21 and 40 mg of dexamethasone on days 1, 8, 15 and 22. In both the groups, patients received only dexamethasone and lenalidomide after the 18th cycle until disease progression.

The primary end point of the study was PFS where the carfilzomib group showed a significant improvement with median 26.3 months compared to 17.6 months in the control group (HR 0.69, *P* = 0.0001). The ORR was 87.1% in the carfilzomib group and 66.7% in the control group (*P* < 0.001). At the time of interim analysis, the combination of carfilzomib plus dexamethasone and lenalidomide showed a significantly improved PFS in patients with relapsed MM and the median OS was not reached in both groups. In the updated analysis for the ASPIRE trial published by David S. Siegel et al. (92),, median OS values of 48.3 months *vs*. 40.4 months were reported for the carfilzomib and control group, respectively (HR 0.79, *P* = 0.0045). The median OS was 11.4 months longer in the carfilzomib group with patients who received one prior line of therapy. In patients who received more than 2 prior lines of therapy, the median OS was 6.5 months longer in the carfilzomib group. Recent *post hoc* analysis of the ASPIRE trial reported a median PFS of 24.4 *vs*. 15.9 months (HR 0.78; 95% CI 0.54 - 1.12) between the carfilzomib group and the control group in frail patients. Median OS for this patient population was 36.4 *vs*. 26.2 months (HR 0.79; 95% CI 0.57 - 1.08) (104). Occurrence of grade 3 or worse AEs was 87.0% *vs*. 83.3% in carfilzomib group and control group respectively and some selected AEs of interest (grade 3 or higher) included acute renal failure (3.8% *vs*. 3.3%), cardiac failure (4.3% *vs*. 2.1%), ischemic heart disease (3.8% vs. 2.3%), hypertension (6.4% *vs*. 2.3%), hematopoietic thrombocytopenia (20.2% *vs*. 14.9%), and peripheral neuropathy (2.8% *vs*. 3.1%). Combination of carfilzomib with dexamethasone and lenalidomide showed an improvement of PFS by 7.9 months along with improved quality of life compared to the reference treatment regimen of dexamethasone plus lenalidomide in patients with relapsed or refractory MM. Findings of the ASPIRE phase III trial led to the FDA approval for the use of carfilzomib with dexamethasone and lenalidomide for the treatment of patients with relapsed MM who received 1 - 3 prior lines of therapy (92, 93).

Rochchi et al. (109), investigated the efficacy and safety of carfilzomib, dexamethasone and lenalidomide therapy in relapsed or refractory MM patients in real world involving 197 patients. Most reported grade 3 or higher AEs included neutropenia (21%), infections (11%) and hypertension (6%). Median PFS was 19.8 months and 1 year OS was 80.6% with an ORR of 88%. Increased PFS and OS rates were observed in patients who received less than two prior line of therapies and overall, this study indicated KRd to be an effective regimen against relapsed or refractory MM outside of clinical studies without the emergence of novel safety concerns (109).

#### 6.2.3 Daratumumab, Carfilzomib and Dexamethasone (DKd)

Frequent use of lenalidomide based regimens in frontline MM therapy has increased the number of lenalidomide refractory patients. Therefore, effective regimens for lenalidomide refractory MM patients are much needed. As a result, the phase Ib (96) study was initiated to evaluate the daratumumab plus carfilzomib and dexamethasone (DKd) in patients with relapsed or refractory MM after 1-3 prior lines of therapy including bortezomib and an IMiD. Lenalidomide refractory patients were also eligible. This was part of an open label, non-randomized, multi-center, multi-arm phase Ib study named EQUULEUS MMY1001, which assessed the effect of daratumumab in combination with a variety of backbone regimens in newly diagnosed and relapsed or refractory MM patients (12). Clinical data from the DKd arm was reported by Chari et al. (96), and the results indicated that DKd was an effective, well-tolerated regimen with deep responses and encouraging PFS in relapsed or refractory MM patients including those who are lenalidomide refractory. A total of 85 patients participated and all received carfilzomib weekly on days 1, 8 and 15 of all the 28 days cycles (initial dose 20 mg/m^2^; 70 mg/m^2^ thereafter) and 40 mg of dexamethasone once a week. Initial daratumumab dose was administered as a single infusion (16 mg/kg on day 1 of cycle) for 10 patients and the other 75 patients received a split fist dose (8 mg/kg on days 1 and 2 of cycle1). Considering the population of patients involved in the study, 95% were lenalidomide exposed and 60% was lenalidomide refractory. Primary endpoints were safety and tolerability of DKd and the secondary endpoints included ORR and OS. After a median follow up of 16.6 months, ORR was 84% and both median PFS and OS were not reached. The 12 months PFS rates were 74% for all treated patients and 65% for lenalidomide refractory patients. The most common grade 3/4 AEs included thrombocytopenia (31%), lymphopenia (24%), anemia (21%), and neutropenia (21%) (96).

Based on the promising data from the DKd treatment arm in EQUULEUS MMY1001 study, a phase III clinical trial named CANDOR (97) was initiated. In this randomized, multicenter, open label study a total of 466 patients were randomly assigned to receive DKd or Kd. Carfilzomib was administered twice weekly for all the patients (20 mg/m^2^ on days 1 and 2 of cycle 1 and 56 mg/m^2^ thereafter). Daratumumab was administered intravenously at a starting dose of 8 mg/kg on days 1 and 2 during cycle 1. Daratumumab dose was increased to 16 mg/kg and administered weekly for the remaining doses for the first 2 cycles, then every 2 weeks for four cycles and every 4 weeks thereafter. All the patients received a weekly dose of 40 mg dexamethasone which was decreased to 20 mg for patients ≥ 75 years old starting from the second week. The primary end point was PFS. After a median follow up of 16.9 months, median PFS was not reached in the DKd arm *vs*. 15.8 months in the Kd arm (HR 0.63; *P* = 0.0027). DKd group showed a significantly prolonged PFS compared to the Kd group with a 37% reduction in the risk of progression or death. The risk of progression or death was reduced in DKd *vs*. Kd across the pre-specified subgroups as well, particularly among lenalidomide refractory and exposed patients.

The Kaplan-Meier 18-month PFS rates were 62% in the DKd group and 43% in the Kd group. ORR was 84% in DKd arm *vs*. 75% in Kd arm (*P*=0.0080). Median treatment duration was longer in DKd compared to Kd (70.1 weeks vs. 40.3 weeks). At a median follow up time of 17.2 months (DKd group) and 17.1 months (Kd group), median OS was not reached in either groups (HR 0.75; *P* =0.17). The Kaplan-Meier 18-month OS rates were 80% in the DKd group and 74% in the Kd group. Grade 3 or higher AEs were reported in 82% of the patients in the DKd group *vs*. 74% in the Kd group. Most commonly observed all grade AEs included thrombocytopenia, anemia, diarrhea, hypertension, upper respiratory infections, fatigue and dyspnea. Grade 3 or worse cardiac failure was 4% in DKd and 8% in Kd. Grade 3 or higher acute renal failure showed a similar occurrence to cardiac failure with 3% in DKd and 7% in Kd arm. AEs leading to treatment discontinuation was 22% in the DKd group and 25% in the Kd group (97). Findings from the CANDOR trial led to the FDA approval of DKd treatment regimen in 2020 for adult patients with relapsed or refractory MM who have received 1 - 3 lines of prior therapy (12).

#### 6.2.4 Isatuximab, Carfilzomib and Dexamethasone (IKd)

Open label, phase III randomized parallel group clinical trial was initiated by Moreau et al. (98), to assess the efficacy of addition of isatuximab to carfilzomib and dexamethasone regimen in treatment of relapsed MM. Isatuximab is an IgG1 monoclonal antibody that kills myeloma cells by targeted binding with an epitope in CD38 (110, 111). In this isatuximab, carfilzomib and dexamethasone in relapsed multiple myeloma (IKEMA) trial, patients with relapsed or refractory MM who received 1 to 3 prior line of therapies were randomly assigned to receive isatuximab, carfilzomib and dexamethasone (isatuximab group) or carfilzomib and dexamethasone (control group) (98). Isatuximab was administered intravenously at 10 mg/kg on days 1,8,15 and 22 on first 28 days cycle and on 1 and 15 days on the subsequent cycles. All the patients received carfilzomib intravenously twice weekly (20 mg/m^2^ on days 1 and 2 of cycle 1 and 56 mg/m^2^ thereafter). Dexamethasone was administered orally or intravenously to patients in both groups (20 mg on days 1, 2, 8, 9, 15, 16, 22, and 23). Primary end point was PFS, and secondary key endpoints included ORR, OS and rate of very good partial response or better. After a median follow up of 20.7 months, PFS was not reached in the isatuximab group compared to the 19.15 months (95% CI 15.77–not reached) in the control group. This was a significant improvement in PFS in the isatuximab group compared to the group receiving carfilzomib dexamethasone combination therapy. (HR 0.53; 99% CI 0·32-0.89; one sided P =0.0007). Both DOR and time to next treatment was longer in isatuximab group compared to the control group and treatment emergent AEs of grade 3 or worse were reported in 77% in isatuximab group *vs*. 67% in the control group. Discontinuation of treatment due to treatment emergent AEs was reported 15% in isatuximab group compared to 17% in the control group. Overall results from this trial showed a significant improvement in PFS and a better depth and quality of response to treatment in patients with relapsed MM by the addition of isatuximab to carfilzomib and dexamethasone regimen. Based on the data obtained from IKEMA trial FDA approval was given to the use of isatuximab, carfilzomib and dexamethasone for adult patients with relapsed or refractory MM who received one to three prior lines of therapies on March 31, 2021 (98, 112).

## 7 Carfilzomib Associated Toxicity

In 2013, Siegel et al. (113) published safety data for single agent carfilzomib for 526 patients with advanced MM who participated in 4 phase II studies PX-171-003-A0, PX-171-003-A1, PX-171-004 and PX-171-005. According to the final report the most common AEs of any grade included fatigue (55.5%), anemia (46.8%) and nausea (44.9%). Overall, occurrence of peripheral neuropathy was low (13.9%). Disregarding disease progression as an AE, the most common serious AEs (SAEs) were pneumonia (9.9%), acute renal failure (4.2%), pyrexia (3.4%) and congestive heart failure (CHF) (3.4%). Dose reduction due to AEs occurred in 77 patients (14.6%) and 119 patients (22.6%) required a dose delay while 14.8% of the patients discontinued treatment due to an AE. Taken together, the study indicated a favorable safety profile for single agent carfilzomib in patients with advanced MM and the general tolerability of carfilzomib observed in the analysis allows the administration of full-dose carfilzomib for extended periods in a wide spectrum of patients with R/RMM (113). Summary of the AEs reported in the integrated safety profile for single agent carfilzomib in patients with advanced MM is shown in **Table 2**.

**Table 2:** Summary of the AEs reported in the integrated safety profile for single agent carfilzomib in patients with advanced MM.

	Single agent carfilzomib integrated safety profile (113) (N = 526)	
Adverse event	All grades (%)	Grade 3 or worse (%)
**Hematological**		
Anemia	46.8	22.4
Thrombocytopenia	36.3	23.4
Lymphopenia	24.0	18.1
Neutropenia	20.7	10.3
Leukopenia	13.5	5.3
**Non-hematological**		
Fatigue	55.5	7.6
Nausea	44.9	1.3
Dyspnea	34.6	4.9
Diarrhea	32.7	1.0
Pyrexia	30.4	1.7
Upper respiratory tract infection	28.3	3.2
Headache	27.6	1.3
Cough	26.0	0.2
Increased serum creatinine	24.1	2.7
Peripheral edema	24.0	0.6
Vomiting	22.2	1.0
Constipation	20.9	0.2
Back pain	20.2	2.9
Pneumonia	12.7	10.5

Considering the completed phase III studies involving carfilzomib in combination with dexamethasone and or lenalidomide against advanced MM, the summarized adverse event profiles for ASPIRE and ENDEAVOR trials are outlined in **Table 3**. Safety data from the ASPIRE trial indicated that the occurrence of grade 3 or worse AEs (87.0% vs. 83.3%) and SAEs (65.3% *vs*. 56.8%) was more frequent in the KRd group compared to the Rd group. Cardiac AEs were also resulted at a higher rate in the KRd group. Treatment discontinuation rates were similar in both groups and no new safety signals related to carfilzomib were observed (92). During the ENDEAVOR trial, incidence of grade 3 or worse AEs, SAEs and fatal AEs were higher in the Kd group compared to the Vd (bortezomib and dexamethasone) group. Grade 3 or worse AEs that resulted at a higher rate in the Kd group included anemia, hypertension, dyspnea, decrease in lymphocyte count, pyrexia, and cardiac failure. Despite having nearly a twice longer median treatment exposure, the exposure adjusted incidence of overall grade 3 or worse AEs and fatal AEs of the Kd group was similar to the Vd group (88). According to a *post hoc* analysis study of carfilzomib combination regimens in the treatment of R/RMM in frail patients involving ASPIRE, ENDEAVOR and A.R.R.O.W trials, efficacy and safety data were consistent in frail patients with the data reported in primary studies and this indicated the carfilzomib combination therapy should not be restricted by the frailty status (104).

**Table 3:** Summary of selected AEs recorded in the safety profiles for ASPIRE and ENDEAVOR trial.

Adverse Events	ASPIRE trial (93)				ENDEAVOR trial (88, 94)			
	KRd group (N = 392) %		Rd group (N = 389) %		Kd group (N = 463) %		Vd group (N = 456) %	
	All grades	Grade ≥ 3	All grades	Grade ≥ 3	All grades	Grade ≥ 3	All grades	Grade ≥ 3
Acute renal failure^1^	8.4	3.3	7.2	3.1	10.4	5.6	6.1	3.3
Anaemia	42.6	17.9	39.8	17.2	42.5	16.4	28.3	10.1
Cardiac failure^2^	6.4	3.8	4.1	1.8	10.8	5.8	3.3	2.0
Constipation	20.2	0.3	17.2	0.5	16.2	0.4	27.6	1.8
Cough	28.8	0.3	17.2	0	27.6	0	15.8	0.2
Diarrhea	42.3	3.8	33.7	4.1	36.3	3.9	40.6	8.6
Dyspnea	19.4	2.8	14.9	1.8	32.2	6.3	13.6	2.2
Fatigue	32.9	7.7	30.6	6.4	32.2	6.7	30.7	7.7
Headache	not given	not given	not given	not given	20.5	0.9	10.7	0.7
Hypertension	14.3	4.3	6.9	1.8	32.2	14.5	9.9	3.3
Hypokalemia	27.6	9.4	13.4	4.9	13.0	2.4	11.1	3.7
Lymphopenia	not given	not given	not given	not given	6.7	4.8	5.5	3.1
Muscle spasms	26.5	1.0	21.1	0.8	19.9	0.2	6.1	0.7
Nausea	not given	not given	not given	not given	23.5	1.9	20.0	0.7
Neutropenia	37.8	29.6	33.7	26.5	6.0	2.4	5.7	2.2
Peripheral Neuropathy	17.1	2.6	17.0	3.1	10.9	1.3	28.5	6.1
Pneumonia	not given	not given	not given	not given	11.4	9.1	11.6	8.6
Pyrexia	28.6	1.8	20.8	0.5	32.4	3.0	15.1	0.7
Thrombocytopenia	29.1	16.6	22.6	12.3	21.6	8.9	18.4	9.4
Upper respiratory tract infection	28.6	1.8	19.3	1.0	25.7	1.7	18.2	0.9

KRd, carfilzomib +Rd; Rd, lenalidomide + dexamethasone; Kd, carfilzomib + dexamethasone; Vd, bortezomib + dexamethasone.

^1^Acute renal failure group includes acute renal failure, renal failure, renal impairment, azotemia, oliguria, anuria, toxic nephropathy, acute pre-renal failure and pre-renal failure.

^2^Cardiac failure group includes cardiac failure, congestive cardiac failure, pulmonary edema, hepatic congestion, cardiopulmonary failure, acute pulmonary edema, acute cardiac failure and right ventricular failure for ASPIRE trial and cardiac failure, ejection fraction decreased, pulmonary edema, acute cardiac failure, congestive cardiac failure, acute pulmonary edema, acute left ventricular failure, chronic cardiac failure, cardiopulmonary failure, hepatojugular reflex, right ventricular failure, and left ventricular failure for ENDEAVOR trial.

### 7.1 Carfilzomib Associated Cardiotoxicity

A systematic review analyzing the published data regarding the use of carfilzomib in 29 eligible clinical trials reported that the occurrence of all-grade cardiotoxicity was 8.68% and high-grade cardiotoxicity was 4.92% (114). These values seem higher compared to the data obtained from clinical trials involving bortezomib based regimens which are 3.8% and 2.3% for all-grade and high-grade cardiotoxicity, respectively (115). During the ENDEAVOR trial, cardiac toxicity of carfilzomib was compared with that of bortezomib. Any cardiac event in any grade was reported in 12% of the patients in the carfilzomib treatment group compared to 4% in the bortezomib treatment group (94). Another systematic review and a meta-analysis of randomized control trials reported a significant increased risk of heart failure in MM patients treated with carfilzomib. They analyzed reported data from randomized phase III trials involving the use of carfilzomib against MM and results indicated 8.1% occurrence of heart failure in the carfilzomib arm compared to the 3.4% in the control arm (116). Patients with coexisting cardiovascular diseases and other comorbidities are at a higher risk of developing cardiotoxicity during carfilzomib treatment which raises the need for a thorough cardiovascular risk assessment process prior to carfilzomib therapy (117). A recent study investigating the molecular mechanism of carfilzomib induced cardiotoxicity in mice indicated that the upregulation of protein phosphatase (PP)-2A activity by carfilzomib and the subsequent inhibition of AMPKα (AMP-activated protein kinase α subunit) mediated autophagy is closely associated with the carfilzomib induced cardiac dysfunction. Additionally, this study indicates the importance of metformin (Met), a potential cardioprotective agent against carfilzomib related cardiotoxicity (118).

### 7.2 Carfilzomib Associated Kidney Toxicity

A systematic review and meta-analysis of randomized control trials involving carfilzomib regimens was performed by Ball et al. (119), to characterize the elevated risk of kidney toxicity in MM patients. In this study, data from four randomized control trials with 2954 patients were analyzed and results indicated a 21.3% cumulative rate of kidney toxicities of all grades in the carfilzomib arm. Cumulative rate of grade 3 - 5 AEs was 8.3% with a significantly increased pooled incidence rate ratio of 1.66 in the carfilzomib arm compared to the control group. Acute kidney injury was reported to be the most common renal AE in this analysis (119). Milan et al. (120), performed an investigation to study the rate of renal failure and associated risk factors utilizing carfilzomib against relapsed or refractory MM in a real world patient population. Renal failure was reported in 22% of the patient population which was higher than the cumulative incidence rate of renal AEs in clinical trials. History of cardiac disease and chronic kidney disease along with increasing age was identified as associated risk factors for renal failure in this study (120).

## 8 Ixazomib: First Oral Proteasome Inhibitor With a Favorable Safety Profile

The development of a new proteasome inhibitor was initiated to overcome the limitations of bortezomib and carfilzomib, to improve efficacy and to develop tolerance towards resistance mechanisms. Ixazomib, a small molecule with structural resemblance of bortezomib was developed as the first clinically available oral proteasome inhibitor approved by the FDA in 2015 based on the Phase III TOURMALINE-MM1 trial and is widely used to treat MM patients who have undergone at least one prior therapy. The oral administration of ixazomib can provide simple and less troublesome proteasome inhibition therapy for many MM patients. Ixazomib can be readily used in Phase III trial programs in newly diagnosed multiple myeloma or RRMM, maintenance therapy in transplant eligible and ineligible patients and for multiple early phase studies (121).

Ixazomib is a citrate ester of boronic acid which is a stable prodrug. At physiological conditions, it is hydrolyzed into a free, biologically active boric acid metabolite (MLN2238) which is an N-capped dipeptidyl leucine boronic acid (122). This active molecule targets mainly the β5 chymotrypsin-like subunits and also binds with β1 caspase-like and β2 trypsin-like subunits of 20S proteasome at higher concentrations (123). The half- life of dissociation of ixazomib from proteasome is six times less from bortezomib which allows easy penetration of the proteasome inhibitor into the tissues and higher recovery of proteasome activity (124).

In all the clinical trials of MM patients, ixazomib is well tolerated as a single agent and in combinations especially with dexamethasone in treatment of R/RMM. Globally, strong anti-myeloma activity and manageable toxicity profiles were observed in Ixazomib treated MM patients in Phase III trials of MM. Some side effects were detected in these global clinical trials of MM patients including rashes, lower number of white blood cells and platelets, fatigue, diarrhea, nausea and peripheral neuropathy (124, 125). A new practice was introduced with the combination of Ixazomib and lenalidomide and dexamethasone (IRd) which is the first oral triplet therapy for multiple relapsed (two or more relapses) patients. This combination showed high response rate and progression-free survival of 17.7 months where TOURMALINE-MM1 clinical trial reported PFS as 20.6 months. This has significant effect in anti-myeloma treatment with multiple relapsed setting when larger population (116 patients) was analyzed. These patients were treated at least one dose of IRd where 79.3% patients received 2 or more prior therapies. Among them 95.7% is treated with PIs initially. However, one third of the patients fail to achieve minimal response in prior therapies. IRd treated patients showed significant response where 64-66% of patients had higher response rate when IRd treatment was received beyond second line (126). Adverse effects were well-manageable with IRd compared to Ixazomib alone. Ixazomib and IRd are promising and highly convenient oral therapy available for MM treatment, mainly for relapsed or refractory MM patients, as a maintenance therapy and a promising therapy for newly diagnosed patients.

## 9 Conclusion

Over the past decades, proteasome inhibitors have gained significant attention as a promising approach to treat R/RMM. Carfilzomib was discovered as a second-generation proteasome inhibitor which overcomes several limitations of the first-generation proteasome inhibitor, bortezomib. Carfilzomib effectively inhibits proteasome activity by forming an irreversible, highly selective complex with proteasome *via* a unique mechanism and has shown enhanced efficacy in the treatment of MM. However, some MM cells show resistance to carfilzomib and toxicity with intravenous administration. Despite having a different toxicity profile to other proteasome inhibitors; carfilzomib is associated with a higher occurrence of cardiovascular AEs. Therefore, assessment of cardiovascular risk factors prior to the initiation of carfilzomib therapy and close monitoring for treatment emergent cardiovascular AEs is necessary in carfilzomib therapy. In addition to cardiotoxicity, Carfilzomib treatment is also associated with renal toxicity. These side-effects continue to challenge the treatment of MM with Carfilzomib and optimization of Carfilzomib treatment regimens to attenuate such effects is a timely need.

## Author Contributions

Conceptualization, editing and supervision, GS. Investigation, writing-review and editing, SJ, SW, and DR. Visualization, SJ and SW. All authors contributed to the article and approved the submitted version.

## Conflict of Interest

The authors declare that the research was conducted in the absence of any commercial or financial relationships that could be construed as a potential conflict of interest.

## Publisher’s Note

All claims expressed in this article are solely those of the authors and do not necessarily represent those of their affiliated organizations, or those of the publisher, the editors and the reviewers. Any product that may be evaluated in this article, or claim that may be made by its manufacturer, is not guaranteed or endorsed by the publisher.

## Abbreviations

AE, Adverse Events; CI, Confidence Interval; DKd, Daratumumab, Carfilzomib and Dexamethasone; DOR, Duration of Response; ER, Endoplasmic Reticulum; HR, Hazard Ratio; Kd, Carfilzomib and Dexamethasone; KRd, Carfilzomib, Lenalidomide and Dexamethasone; MCL, Mantle Cell Lymphoma; MM, Multiple Myeloma; ORR, Overall Response Rate; PFS, Progression Free Survival; RRMM, Relapsed and Refractory MM; R/RMM, Relapsed and/or Refractory MM; SAE, Serious Adverse Events; UPP, Ubiquitin Proteasome Pathway; UPR, Unfolded Protein Response.
